# Colloidal Silver in Rheumatism

**Published:** 1911-06

**Authors:** 


### Colloidal Silver in Rheumatism

Writing in the *Medizinische Klinik* of October 31, 1909, Dr. Michael Spitzer, Assistant to Dr. Singer at the first medical ward of the Imperial Hospital "Rudolfstiftung" in Vienna, says: Fully convinced that articular rheumatism, in the majority of cases, constitutes a pyemic process, Dr. Singer has for more than ten years used collargolum and unguentum Credé in the treatment of this disease. Unguentum Credé may be applied locally to the affected parts, or used percutaneously over a larger area, like mercuric ointment. Collargol is best used in form of enemata, 7½ up to 75 grains in 3 to 6 ounces of distilled water being given. In a great number of cases where the smaller joints were affected, excellent results have been obtained, although at times the remedy failed to produce improvement. Various other therapeutic agents are used at the hospital in the treatment of articular rheumatism, but in obstinate cases Dr. Singer, both in his private practice and at the hospital, always falls back upon argentum colloidal, giving preference to the Credé preparations.

F. I. Gramenitzkij describes in "Russkij Wratsch" thirteen cases of cystitis of greatly varied pathogenesis. Nearly all of them were successfully treated by cleansing the bladder with a 3 per cent. solution of boric acid, followed by intravesical injections of 100 c.c. of a 1 per cent. collargol solution which most of the patients were able to retain until the next micturition. Only in two cases of chronic cystitis, one of gonorrheal origin, and one due to prostatic hypertrophy, the patients could not retain more than 50 c.c. of the collargol solution. In one case of tubercular cystitis, in which both kidneys were involved, the result was negative, while in another similar case, in which only one kidney was involved, collargol, after removal of the latter, exercised a very beneficial influence upon the tubercular infection of the bladder. The author considers collargol a valuable therapeutic agent in cystitis, whenever the process is limited to the bladder.

